# A novel 8-connected Pixel Identity GAN with Neutrosophic (ECP-IGANN) for missing imputation

**DOI:** 10.1038/s41598-024-73976-7

**Published:** 2024-10-13

**Authors:** Gamal M. Mahmoud, Mostafa Elbaz, Fayez Alqahtani, Yasser Alginahi, Wael Said

**Affiliations:** 1 Department of Electrical Engineering, Pharos University in Alexandria, Alexandria, Egypt; 2 Department of Computer Science, Faculty of Computers and Informatics, Kafrelsheikh University, Kafrelsheikh, Egypt; 3 Software Engineering Department, College of Computer and Information Sciences, King Saud University, Riyadh, 12372 Saudi Arabia; 4 Department of Computer Science, Adrian College, Adrian, Michigan USA; 5 Computer Science Department, Faculty of Computers and Informatics, Zagazig University, 44511 Zagazig, Egypt

**Keywords:** GANs, Missing pixel imputation, Data imputation, Mode collapse, Missing pixel, Neutrosophic, Identity block, Mathematics and computing, Computer science

## Abstract

Missing pixel imputation presents a critical challenge in image processing and computer vision, particularly in applications such as image restoration and inpainting. The primary objective of this paper is to accurately estimate and reconstruct missing pixel values to restore complete visual information. This paper introduces a novel model called the Enhanced Connected Pixel Identity GAN with Neutrosophic (ECP-IGANN), which is designed to address two fundamental issues inherent in existing GAN architectures for missing pixel generation: (1) mode collapse, which leads to a lack of diversity in generated pixels, and (2) the preservation of pixel integrity within the reconstructed images. ECP-IGANN incorporates two key innovations to improve missing pixel imputation. First, an identity block is integrated into the generation process to facilitate the retention of existing pixel values and ensure consistency. Second, the model calculates the values of the 8-connected neighbouring pixels around each missing pixel, thereby enhancing the coherence and integrity of the imputed pixels. The efficacy of ECP-IGANN was rigorously evaluated through extensive experimentation across five diverse datasets: BigGAN-ImageNet, the 2024 Medical Imaging Challenge Dataset, the Autonomous Vehicles Dataset, the 2024 Satellite Imagery Dataset, and the Fashion and Apparel Dataset 2024. These experiments assessed the model’s performance in terms of diversity, pixel imputation accuracy, and mode collapse mitigation, with results demonstrating significant improvements in the Inception Score (IS) and Fréchet Inception Distance (FID). ECP-IGANN markedly enhanced image segmentation performance in the validation phase across all datasets. Key metrics, such as Dice Score, Accuracy, Precision, and Recall, were improved substantially for various segmentation models, including Spatial Attention U-Net, Dense U-Net, and Residual Attention U-Net. For example, in the 2024 Medical Imaging Challenge Dataset, the Residual Attention U-Net’s Dice Score increased from 0.84 to 0.90, while accuracy improved from 0.88 to 0.93 following the application of ECP-IGANN. Similar performance enhancements were observed with the other datasets, highlighting the model’s robust generalizability across diverse imaging domains.

## Introduction

Missing pixel imputation is critical in image processing and computer vision, and it encompasses various applications such as image restoration and inpainting^1–4^. The process involves estimating and filling in missing values in images, thereby completing its visual information. Accurate pixel imputation is crucial for preserving image integrity and quality, enabling subsequent analysis and interpretation^5^.

Missing pixel imputation is crucial for image restoration and plays a significant role in fields such as image label denoising^6^. In scenarios where training datasets suffer from noisy labels—often due to misclassifications or inconsistencies—accurate imputation of missing pixel values can enhance the overall quality of the dataset. For example, the method “Cross-to-merge training with class balance strategy for learning with noisy labels” utilizes imputation techniques to mitigate the effects of label noise, thereby improving the model’s ability to learn from cleaner data. Ensuring that the imputed pixels align well with the surrounding context aids in generating more reliable training samples. Additionally, “A joint end-to-end framework for learning with noisy labels” incorporates missing pixel imputation to refine the learning process, allowing models to handle better the uncertainties associated with noisy labels. These frameworks demonstrate that addressing missing pixel inputs enhances images’ visual fidelity and contributes significantly to the robustness and accuracy of machine learning algorithms in diverse applications such as object detection and image classification. By recognizing the multifaceted applications of missing pixel imputation, we can appreciate its greater importance than traditional image processing tasks^7^.

Imputing missing pixels entails the exploration of various methodologies to estimate and fill in gaps in an image effectively. However, many methods have introduced impute pixels, such as traditional approaches like nearest neighbour interpolation or simple statistical techniques. However, these methods often fail to capture an image’s complex dependencies and contextual information^8,9^. Consequently, the imputed pixels may require more fidelity and coherence with the surrounding content, resulting in visually unconvincing results. In addition, these methods need help to handle challenging scenarios such as occlusions, irregular patterns, and varying image structures. Researchers have investigated advanced techniques, including machine learning-based approaches and deep neural networks, to overcome these limitations and achieve more accurate and realistic missing pixel imputation.

In recent years, Generative Adversarial Networks (GANs) have emerged as a promising approach to address the challenges of missing pixel imputation. GANs leverage the power of deep learning and adversarial training to learn the data distribution and generate visually coherent and plausible missing pixel predictions^10–14^. GANs have garnered significant attention in computer vision tasks due to their remarkable ability to create realistic, high-quality images^15^. GANs have two main components: a generator network that attempts to produce synthetic samples and a discriminator network that distinguishes between actual and generated samples. The GANs learn to capture the underlying data distribution and generate visually convincing outputs by training these adversarial networks. In the context of missing pixel imputation, GANs offer a promising alternative to traditional methods by leveraging their generative capabilities to fill in missing values in an image^16^.

However, applying GANs to missing pixel imputation is challenging. Two primary issues arise when mode collapse, where the generator fails to explore the full diversity of possible missing pixel values because the model gets stuck in generating the same pre-generated pixel; this takes a large number of trials to reach the stopping criteria or until to come to the error tolerance value, add to this; the generation of pixel values that do not align well with the surrounding context or the generated pixel does not integrate with other parts of the images. The other issue with GANs is that the generator requires many iterations to reach the value of the discriminator. The generator produces a non-accurate pixel if there is any distortion in the discriminator value. Addressing these challenges is critical for ensuring accurate and coherent missing pixel imputation results^17–20^.

This paper proposes a novel approach for missing pixel imputation called 8-connected Pixel Identity GAN with Neutrosophic (ECP-IGANN). The proposed approach builds upon the foundation of GANs while introducing two essential modifications to enhance the imputation process. Firstly, we incorporate an identity block within the pixel generation process, preserving the existing pixel values and ensuring consistency with the original image. Second, we introduce a novel approach to evaluate the generated pixel values by comparing them with the weighted average of the 8-connected pixels surrounding the missing pixel and the actual value of the discriminator. This evaluation allows us to accept or reject the generated pixels based on the degree of error, effectively enhancing the coherence and accuracy of the imputed pixels.

The main contributions of this paper are summarized as follows. Firstly, we address the challenges of missing pixel imputation by proposing a novel 8-connected Pixel Identity GAN with Neutrosophic (ECP-IGANN). The proposed method leverages the power of Generative Adversarial Networks (GANs) while introducing two essential modifications to enhance the imputation process.

- Incorporating an identity block into the ECP-IGANN framework mitigates the mode collapse phenomenon, enabling the generator to produce a diverse range of pixel values rather than repetitive outputs. This enhancement is crucial for ensuring the variability in the generated pixels, which is essential for high-quality image synthesis.
- ECP-IGANN introduces a novel loss function called neutrosophic 8-connected pixel loss, designed to uphold the spatial integrity among pixels in the generated images. This loss function leverages the principles of neutrosophic logic, allowing for a more nuanced evaluation of pixel relationships and improving the coherence of the imputed regions.
- The architecture of ECP-IGANN permits the generation of pixel values without a discriminator, thereby simplifying the computational process. This design choice facilitates the integration of generated pixels with their surrounding context using fewer computational resources and a reduced number of iterations, making the model particularly advantageous for real-time and low-latency applications where computational efficiency is paramount.
- Comprehensive experiments conducted on different new datasets validated the efficacy of the proposed ECP-IGANN method in terms of diversity and missing pixel imputation. The results reveal that ECP-IGANN not only generates diverse and accurate imputed pixels but also enhances the segmentation results for different architectures of U-Nets.

This paper is organized as follows. Section 2 provides a comprehensive review of related work on missing pixel imputation, including different recent versions of GANs and their advantages and disadvantages. Section 3 presents our proposed model, 8-connected Pixel Identity GAN with neutrosophic (ECP-IGANN), and details the modifications we introduce for enhancing the imputation process, including the incorporation of an identity block and the evaluation of generated pixel values using the weighted average of surrounding pixels. Section 4 describes the experimental setup, including the datasets used and evaluation metrics used to assess the performance of ECP-IGANN. Finally, in Sect. 5, we provide concluding remarks, summarizing the contributions of the proposed method and discussing potential avenues for future research in the field of missing pixel imputation.

## Related work

Missing pixel imputation refers to estimating and reconstructing missing values in an image to restore the image as accurately as possible. It is a specialized subset of missing data imputation where the challenge lies in predicting the absent pixel values by leveraging available information from the surrounding image regions. The critical challenge is to ensure that the imputed pixels are not only visually plausible but also consistent with the underlying structure and content of the image. This process is pivotal in image restoration and analysis across various domains, as it enables the replacement of missing values with statistically or algorithmically estimated substitutes.

Recent advances in deep learning and image processing have significantly enhanced the accuracy and efficiency of imputation methods, yielding superior results in medical imaging^20^, remote sensing^21^, video restoration^22^, and digital art restoration^23^. Modern techniques for missing pixel imputation are predominantly based on deep learning algorithms, including autoencoders such as adversarial networks (GANs)^24,25^.

In the literature, various GAN-based approaches for missing pixel imputation have been proposed to improve the accuracy and quality of the imputed pixels. Each GAN variant has distinct advantages and limitations; these limitations can lead to inaccurately generated pixels or require numerous iterations to achieve satisfactory results. GAN-based imputation methods are trained on datasets containing images with missing pixels, enabling the model to learn the data’s underlying patterns and structural characteristics. The generator network synthesizes new pixel values, which are subsequently evaluated against the original image by the discriminator network. This adversarial training process iterates until the generated pixels closely approximate the original missing values, resulting in a reconstructed image with minimal errors.

Yoon et al.^26^ introduced the Generative Adversarial Imputation Networks (GAIN) framework in 2018, leveraging the Generative Adversarial Network (GAN) paradigm to address the challenge of missing data imputation. The core concept of GAIN is to generate imputed data that closely match the observed portions of an image using an element-wise discriminator. This approach ensures that the imputed values are indistinguishable from the original data, enhancing the restored image’s overall fidelity. GAIN was empirically validated on five open-source datasets—Breast, Spam, Letter, Credit, and News—sourced from the UCI Machine Learning Repository, where it outperformed existing techniques in terms of imputation accuracy for data missing completely at random (MCAR). Despite these advancements, the GAIN framework, like many GAN-based approaches, faces limitations. One notable disadvantage lies in the integrity of the pixel relationships in the generated images. While GAIN attempts to preserve the local structure, the global coherence between pixels may be compromised, leading to inconsistencies or artifacts that detract from the image’s overall quality.

Moreover, the stability of the GAIN training process is a critical concern. GANs are inherently prone to instability during training, often requiring careful tuning of hyperparameters and network architectures. Another significant drawback is the risk of mode collapse, a common issue in GANs where the generator produces a limited variety of outputs, failing to capture the full diversity of the data distribution.

Daniel et al.^27^ recently contributed to this field by introducing an image inpainting model based on the Wasserstein Generative Adversarial Imputation Network (WGAIN). The generator network in this model employs convolutional layers with varying dilation rates and incorporates skip connections to enhance the reproduction of fine details in the output. This architecture allows the model to serve as a universal imputation tool, capable of handling diverse missingness scenarios with high quality. The model was experimentally validated for three distinct missingness scenarios: randomly missing pixels, various smaller square regions, and a single large square centrally located in the image. The proposed model demonstrated superior inpainting results in all scenarios, as evaluated using peak signal-to-noise ratio (PSNR) and structural similarity index (SSIM) on two real-world benchmark datasets, CelebA faces and Paris StreetView. The performance was compared favourably against biharmonic imputation and several state-of-the-art image-inpainting methods. However, like many GAN-based approaches, the WGAIN model has limitations. A primary concern is mode collapse, a prevalent issue in GAN frameworks where the generator may produce a limited range of outputs. The mode collapse can reduce the diversity in the imputed data, potentially limiting the model’s effectiveness in handling a broad array of missingness patterns. Another disadvantage lies in the integrity of the pixel relationships in the generated images. Although the model is designed to reproduce fine details, there can be inconsistencies in the global coherence of pixel arrangements, especially in complex images. Finally, model stability during training presents a significant challenge. GAN-based models, including WGAIN, are known for their susceptibility to instability, often requiring precise tuning of hyperparameters and network structures to achieve convergence.

Lee et al.^28^ introduced a novel approach that extends the field of image imputation by leveraging Generative Adversarial Networks (GANs). Their method addressed the inherent complexity of natural images, which has challenged traditional imputation techniques. By employing a dual-network framework, Lee et al. effectively captured the intricate relationships between pixels and improved the quality of imputed images. They emphasized the importance of maintaining spatial coherence and contextual integrity and successfully mitigating common issues, such as mode collapse and training instability, in existing GAN-based methods. However, despite these advancements, the proposed method still faces limitations, including difficulties preserving pixel integrity, which sometimes results in artifacts or unnatural transitions in the imputed areas. In addition, mode collapse remained a concern, where the generator could produce a limited variety of outputs, reducing the diversity of the imputed images. Reliance on extensive training data also hindered performance in scenarios with limited available data, impacting the overall effectiveness of the imputation process.

Shange et al.^29^ introduced a novel view imputation approach based on generative adversarial networks (GANs), termed VIGAN. The proposed method first treated each view as a separate domain, identifying domain-to-domain mappings through a GAN using randomly sampled data from each view. Subsequently, a multimodal denoising autoencoder (DAE) was employed to reconstruct the missing view from the GAN outputs based on paired data across the views. By jointly optimizing the GAN and DAE models, the models facilitate knowledge integration for domain mappings and view correspondences, effectively recovering the missing view. Empirical results on benchmark datasets validated the proposed VIGAN approach, demonstrating its superiority over state-of-the-art methods.

Furthermore, the evaluation of VIGAN in a genetic study of substance use disorders underscored its effectiveness and usability in life sciences. Despite these contributions, the VIGAN approach has several limitations. One significant drawback was the potential for mode collapse, where the GAN could produce a restricted range of outputs, limiting the reconstructed views’ diversity. In addition, the reliance on paired data across views could be a constraint in scenarios where such data is sparse or unavailable, impacting the model’s generalizability. Furthermore, the complexity associated with optimizing both the GAN and DAE introduced challenges in training stability, which could affect the overall performance of the imputation process.

Kang et al.^30^ proposed a cross-modal generative adversarial network (CM-GAN) to address the issue of incomplete data missing (CDM) in multimodal data fusion analysis, particularly in environments with communication failures or cyberattacks. Their approach aims to generate long-term time-series data from widely existing spatio-temporal modal data and impute missing values by replacing them with generated data. The proposed CM-GAN demonstrated superior performance on a photovoltaic (PV) power output dataset, outperforming baseline models and achieving state-of-the-art results. They conducted extensive ablation studies to validate the contribution of the cross-modal data fusion technique and the reasonableness of the parameter settings. However, CM-GAN has several disadvantages. The model exhibited challenges related to mode collapse, where the generator produced less diverse outputs, thereby compromising the stability of the training process. In addition, the integrity between imputed pixels was sometimes compromised, leading to inconsistencies in the generated data that could affect downstream tasks.

Yoon et al.^31^ (GAMIN) proposed an imputation architecture designed to address scenarios with extreme levels of missing data, specifically those with over an 80% missing rate. Their approach generates multiple candidate imputations and integrates a confidence prediction mechanism, significantly outperforming traditional methods in handling high-dimensional and complex image data. The proposed GAN-based architecture effectively offered robust statistical inference and managed varying degrees of missing data. Similarly, Alharbi et al. introduced two GAN-based methods for imputing missing numerical datasets, where the imputed values are determined using Euclidean distance. These methods outperformed conventional imputation techniques and maintained robustness across missing data scenarios. However, both approaches face challenges related to mode collapse, where the diversity of generated imputations is compromised, leading to less output variability. In addition, issues related to the stability of the training process and the integrity between imputed pixels were noted, potentially impacting the consistency and accuracy of the imputed data.

Luo et al.^32^ addressed the challenge of missing value imputation by treating it as a data generation problem. Drawing inspiration from the success of Generative Adversarial Networks (GANs) in image generation, they proposed a method to learn the overall distribution of a multivariate time-series dataset using GANs, which could then be utilized to generate missing values for each sample. Unlike image data, time series data are often incomplete due to the nature of the data recording process. The authors employed a modified Gated Recurrent Unit (GRU) within the GAN framework to model the temporal irregularity of incomplete time series. Their experiments on two multivariate time series datasets demonstrated that the proposed model outperformed baseline methods regarding imputation accuracy. In addition, the experimental results revealed that a simple model applied to the imputed data achieved state-of-the-art performance on prediction tasks, highlighting the effectiveness of their approach in downstream applications. However, the method encountered challenges with mode collapse, where the diversity of the generated imputations was limited, reducing the variety and reliability of the generated data. In addition, training stability and the integrity between imputed values were observed, potentially affecting the consistency and overall quality of the imputation results.

The related work methods often struggle with issues such as mode collapse, limited diversity in generated pixels, and poor spatial coherence between the imputed and surrounding pixels. These approaches tend to create results that do not integrate well with their context, leading to visually unconvincing outputs and increased computational costs due to numerous iterations and adjustments.

## Proposed model

This section presents the methodology of ECP-IGANN. The model solves the problems mentioned in related works, such as image integrity, mode collapse, and real-time. ECP-IGANN has three main parts: a generator to generate the missing part of the image, a discriminator to take the actual value as input, and an 8-connected neutrosophic part to calculate the neutrosophic weighted average for the 8-connected pixels. We integrate the identity block with the generator to guarantee the diversity of the generated pixels.

The ECP-IGANN (8-connected Pixel Identity GAN with Neutrosophic) model addresses the challenges of missing pixel imputation, particularly issues related to mode collapse and maintaining the spatial coherence of generated images. The architecture of ECP-IGANN consists of three main components: a generator, discriminator, and novel neutrosophic loss function. The generator creates missing pixel values by leveraging an identity block that preserves the essential features of the input image, ensuring that the generated pixels are consistent with the original image. The discriminator evaluates the generated images against authentic images, distinguishing between real and fake pixels, to guide the generator in producing more accurate and coherent outputs.

A key innovation of the ECP-IGANN model is integrating an identity block into the generator. This identity block allows the model to maintain critical spatial information from the input image while generating new pixel values. By incorporating both 1 × 1 and 3 × 3 convolutional layers, the identity block captures the image’s local and global features. The 1 × 1 convolutions help maintain the spatial dimensions, ensuring that critical relationships between neighbouring pixels are preserved, while the 3 × 3 convolutions focus on extracting finer details. This dual approach allows the generator to produce more diverse and accurate pixel values, effectively mitigating the mode collapse problem common in traditional GANs.

Another significant component of the ECP-IGANN model is the neutrosophic-based 8-connected pixel loss function. The loss function is designed to ensure the spatial coherence of the generated pixels by considering the relationships between each pixel and its eight connected neighbours. The neutrosophic approach evaluates the generated pixels’ truth, indeterminacy, and falsehood values, providing a more nuanced assessment of the generated images. By comparing these values with those from the actual images, the loss function ensures that the generated pixels are accurate and contextually consistent with their surrounding pixels, thereby maintaining the overall integrity of the image.

The training process of the ECP-IGANN model involves a feedback loop in which the loss calculated by the discriminator is used to update the generator’s parameters. The goal is to minimize loss and guide the generator to produce images that are indistinguishable from real ones. The model employs a stopping criterion based on the convergence of the generated and actual pixel values, which is determined by the neutrosophic loss function. This approach allows the model to focus on areas where the generated image significantly deviates from the actual image, leading to more efficient and targeted training. An identity block stabilizes the training process by ensuring adequate gradient flow, which is crucial for robust learning dynamics.

### Methodology block diagram

**Fig. 1:**
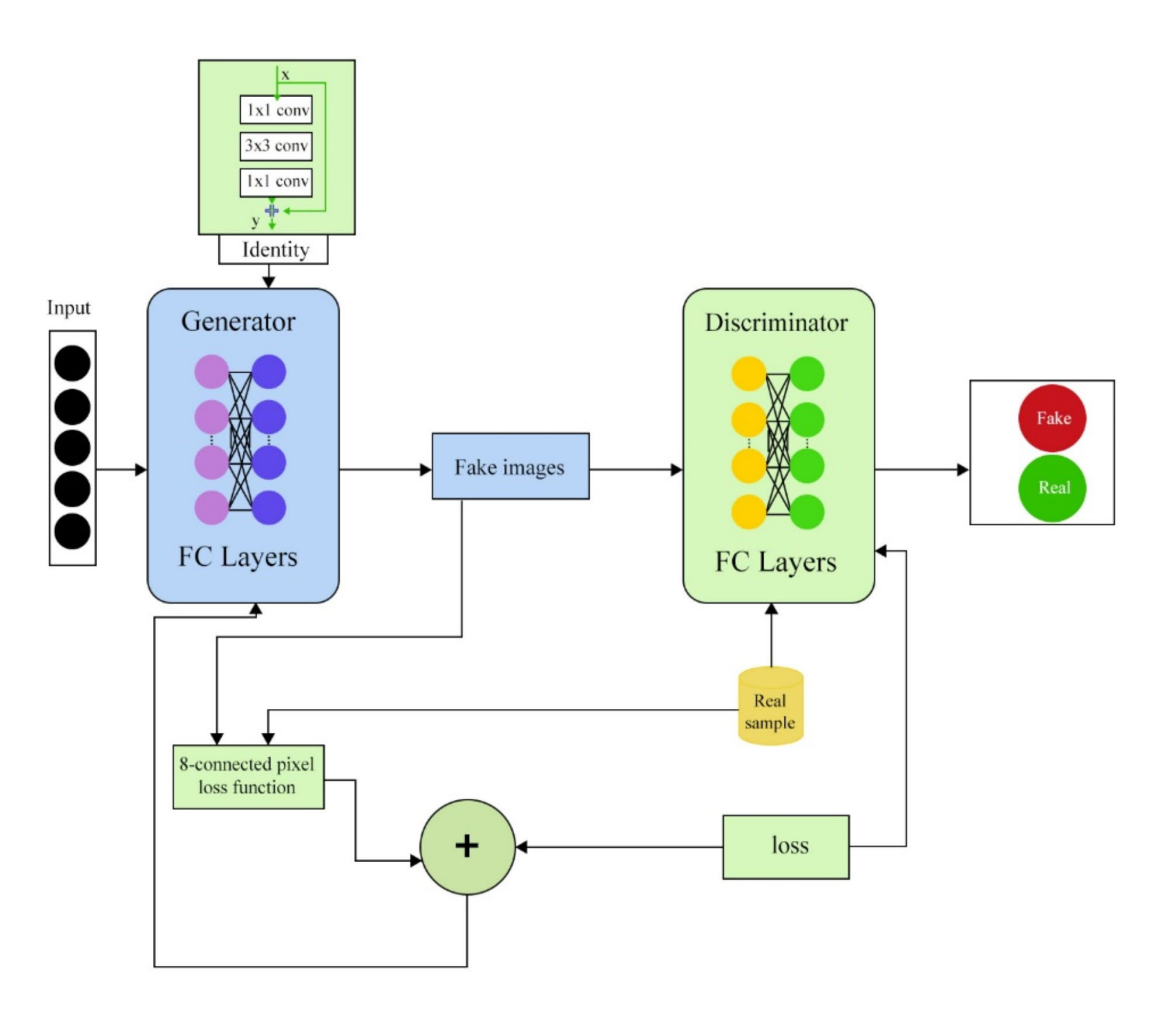
Block diagram of the ECP-IGANN framework.

As shown in Fig. 1, The diagram illustrates a specialized Generative Adversarial Network (GAN) architecture designed for missing image pixel imputation. The process begins with an input image with missing or corrupted pixels, represented by black dots on the left side of the diagram. The goal is to accurately reconstruct missing pixel information to restore the original image. The first step in the process involves an Identity Block, which processes the input image through a series of convolutional layers, including 1 × 1 and 3 × 3 convolutions. The purpose of this block is to extract essential features from images while preserving its spatial structure. Combining different convolutional filters allows the network to capture local and broader contextual information, which is crucial for understanding the surrounding areas of the missing pixels. Next, the extracted features are fed into the generator. The generator is a neural network depicted in the diagram as a series of Fully Connected (FC) layers. The generator predicts missing pixels by leveraging the learned features from the input image. It outputs a “fake image,” a reconstruction of the original image with the missing pixels filled in. The quality of the reconstructed image is critical, as the generator aims to produce an indistinguishable image from a real, fully intact one. Once the generator produces a fake image, it is passed to the discriminator. The discriminator is another neural network composed of fully connected layers, and its task is to evaluate the authenticity of the images it receives. The discriminator is fed both real images (complete and without missing pixels) and fake images generated by the generator. Its goal is to correctly classify these images as “Real” or “Fake.” This adversarial setup creates a competitive dynamic where the generator improves its reconstruction ability, and the discriminator enhances its ability to detect fakes.

The process involves a Loss Function that is critical in training the Generator and Discriminator. The loss function incorporates an “8-connected pixel loss function,” which accounts explicitly for the spatial continuity around each pixel. In other words, this loss function considers the context of each pixel relative to its neighbours, ensuring that the imputed pixels blend seamlessly with the surrounding areas. This approach is essential for image imputation tasks, where the preservation of spatial coherence directly impacts the visual quality of the reconstructed image. A threshold parameter in the 8-connected pixel loss function measures the similarity between the imputed and actual pixels, which falls below a certain level. The threshold of the loss function indicates that the imputed pixels are very similar to the real ones.

There is a Feedback Loop where the loss calculated from the discriminator’s output updates the generator’s parameters. The generator is trained to minimize this loss and strives to produce images that the discriminator cannot distinguish from real ones. As training progresses, the generator becomes increasingly adept at filling in the missing pixels while the discriminator continually refines its ability to distinguish real and fake images.

The algorithm (1) shows the critical steps in the ECP-IGANN (8-connected Pixel Identity GAN with Neutrosophic) model for missing pixel imputation. The model begins by initializing the vital parameters, including the generator, discriminator, loss functions, and learning rates. The training process is then described, where the model iterates through each epoch, generating fake images, evaluating them against real images, and calculating the corresponding losses. The discriminator is updated accordingly, and if the pixel loss is below a specified threshold, fine-tuning is applied to the generator. The process includes checks for early stopping to ensure efficient convergence. After training, the generator and discriminator models are saved, and the final step involves using the trained generator to impute missing pixels in the input images. The imputed images are then returned as the output of the model. This pseudocode provides a clear and structured approach to implementing the ECP-IGANN model, highlighting the essential phases of initialization, training, model saving, and image imputation.

### Neutrosophic 8-Connected pixel loss function

This section presents a method for calculating the loss in a Generative Adversarial Network (GAN). This section presents a method to calculate the loss in a Generative Adversarial Network (GAN) using a novel approach based on neutrosophic sets. By associating each pixel value with corresponding membership values for truth (T), indeterminacy (I), and falsehood (F), we can enhance the representation of pixel intensity, thereby allowing for more nuanced comparisons between generated and real images. The proposed process incorporates an 8-connected pixel neighbourhood comparison, which enables the GAN to consider spatial relationships and improve the quality of generated images.

In Table (1), each pixel value from 0 to 255 is associated with a corresponding neutrosophic set. The membership values of the truth (T), indeterminacy (I), and falsehood (F) components can be adjusted according to requirements. The neutrosophic uses Eqs. (1), (2), and (3) to convert each pixel value into a neutrosophic set.

1 $$\:T=1-\frac{Pixe{l}_{value}\:}{ma{x}_{value}}$$

2 $$\:I=\left(\frac{Pixe{l}_{value}\:}{ma{x}_{value}}\right)*\left(1-\frac{Pixe{l}_{value}\:}{ma{x}_{value}}\right)$$

3 $$\:F=\frac{Pixe{l}_{value}\:}{ma{x}_{value}}$$

**Table 1:** Pixel value representation within a Neutrosophic Set Framework.

Pixel Value	T	I	F
0	1	0	0
1	0.996	0.0039	0.0039
2	0.992	0.0077	0.0078
3	0.988	0.0115	0.0118
4	0.984	0.0152	0.0157
5	0.980	0.0189	0.0196
…	…	…	…
254	0.001	0.2540	0.9961
255	0	0	1


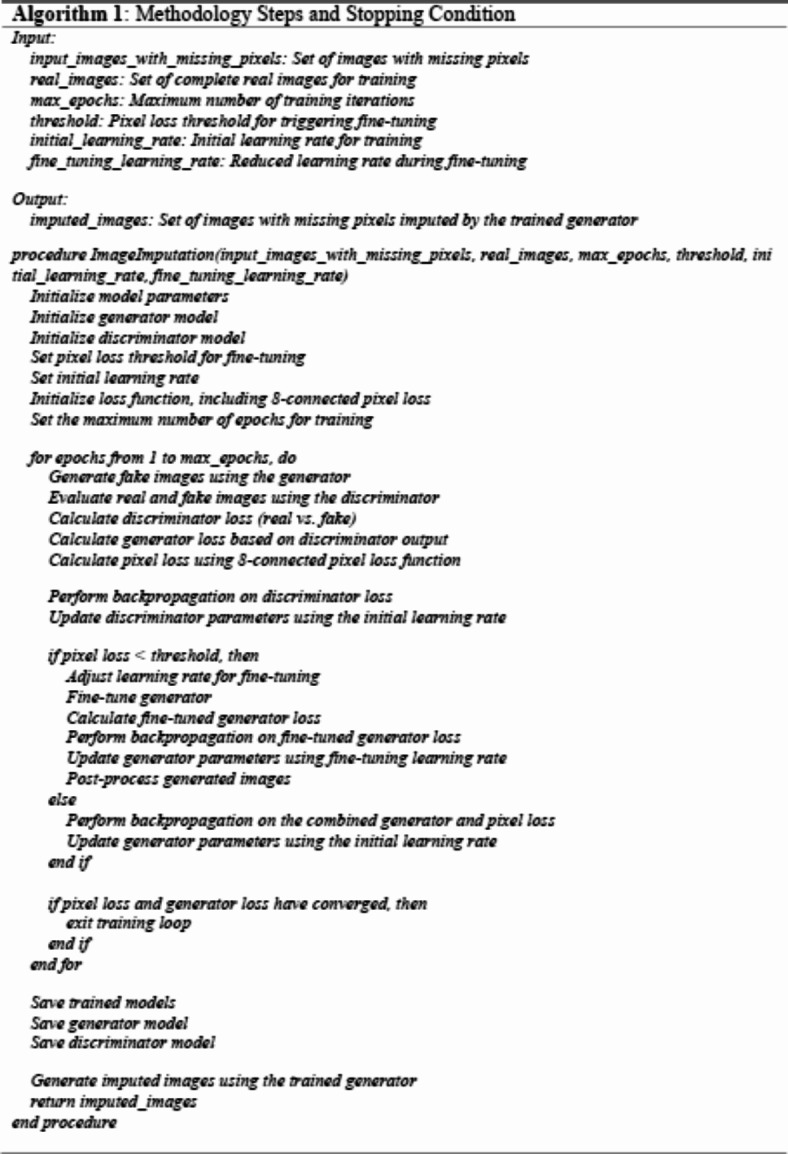


Algorithm (2) presents a method to calculate the loss in a generative adversarial network (GAN) by comparing the 8-connected pixel neighbourhoods in both generated and real images. The process begins by iterating over each pixel in the generated image and identifying its 8-connected neighbourhood and the corresponding neighbourhood in the real image. Each pixel in these neighbourhoods is converted into a neutrosophic set consisting of three components: True (T), Indeterminate (I), and False (F). These components represent different levels of certainty regarding the pixel intensity.

After conversion, the algorithm calculates the average values of these components across the entire neighbourhood for both generated and real images. The critical aspect of this method is the comparison of these neutrosophic averages. Suppose the difference between the averages of the generated and real neighbourhoods is smaller than a specified threshold across all components (T, I, and F). In that case, the algorithm stops further loss calculation for that pixel, considering it sufficiently close to the real pixel. If the differences exceed the threshold, the algorithm calculates the loss based on the squared differences between the neutrosophic components of the generated and real neighbourhoods. This loss is then added to the total loss of the image.

This approach ensures that GAN considers individual pixel accuracy and spatial relationships within an image, thereby encouraging the generation of visually and structurally coherent images. Using neutrosophic sets allows for a more flexible representation of pixel values, thus accounting for uncertainty and indeterminacy. The stopping criterion based on neutrosophic averages helps optimize the learning process, focusing on areas where the generated image deviates significantly from the real image, leading to more efficient and targeted training.


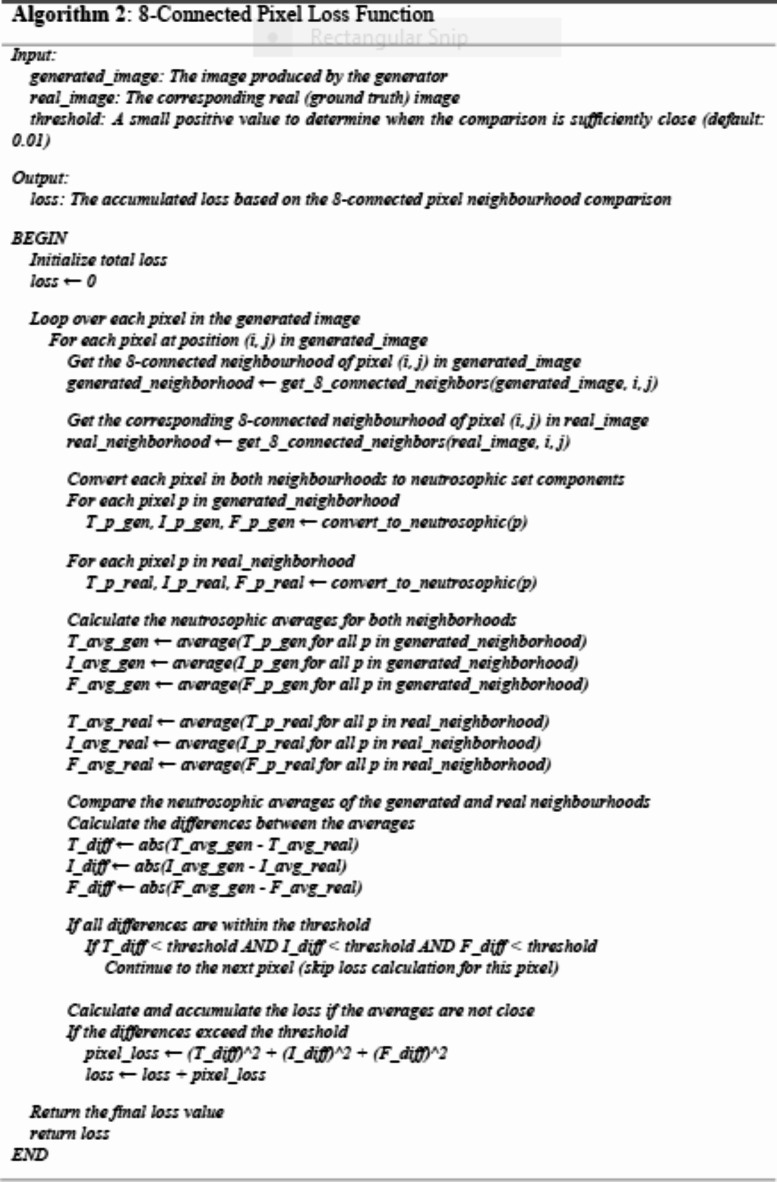


### Identity block in GANs

In the 8-connected Pixel Identity GAN with Neutrosophic (ECP-IGANN), the identity block plays a central role in enhancing the model’s ability to perform missing pixel imputation. The identity block is strategically positioned within the generator network in the 8-connected Pixel Identity GAN architecture with Neutrosophic (ECP-IGANN). Typically, it follows the initial convolutional layers responsible for feature extraction from the input image. By placing an identity block at this juncture, we ensure the generator can effectively integrate the raw pixel values and the learned features extracted from the image. This placement enriches the generated output while maintaining the spatial integrity of the original image. The output from the identity block is then fed into subsequent layers of the generator, facilitating coherent pixel predictions that closely align with the surrounding context. This careful integration enhances the model’s ability to produce diverse and high-quality reconstructions, making it a pivotal element in the overall architecture of ECP-IGANN.

The process begins with the input image, which may contain missing or corrupted pixels represented as black dots. The image is first passed through an identity block, where it undergoes feature extraction through a series of convolutional layers. The 1 × 1 and 3 × 3 convolutional layers within the identity block work in tandem to capture both local and global features of the input. The 1 × 1 convolutions help maintain the spatial dimensions while allowing for learning essential pixel relationships. In contrast, the 3 × 3 convolutions focus on detecting finer details and contextual patterns in images. This combination ensures the network comprehensively understands the image structure^33,34^. After feature extraction, the identity block employs a skip connection that adds the original pixel values to the processed output. This integration ensures that critical information is not lost and preserves image integrity during generation. The enriched features are then forwarded to the generator, which leverages this information to predict and fill in missing pixels accurately.

In addition, the identity block contributes to the overall stability of training by facilitating adequate gradient flow. This mitigates the vanishing gradient problem frequently encountered in GANs, allowing for more robust learning dynamics. By enhancing pixel diversity and coherence, the identity block significantly improves the generator’s capability to produce high-quality and realistic images, thereby addressing the challenges of missing pixel imputation.

A vital feature of an identity block is its skip connection, which allows the original input to bypass specific layers and be added directly to the output. This mechanism ensures that critical pixel values are preserved and maintains visual coherence in the generated images. During processing, the input image flows through an identity block, where the convolutional layers extract relevant features while enriching the data with learned information. The combination of features is then fed into the generator, which uses this enriched data to predict and fill in missing pixels, creating a visually plausible image.

The identity block offers several benefits, including preserving critical information from the input, improved training stability, and enhanced pixel diversity. Allowing gradients to flow more effectively mitigates the risk of mode collapse, a common issue in traditional GAN architectures. The result is a generator that can produce various outputs, ultimately improving the quality of imputed images. Overall, the identity block is pivotal in addressing missing pixel imputation challenges and significantly contributes to the effectiveness of ECP-IGANN in generating high-quality image reconstructions.

### Evaluation metrics

In this paper, we use Eqs. (4), (5), (6), and (7) to calculate four different metrics: RMSE^35^, PSNR^36^, IS^37^, and FID^38^. The RMSE ensures accurate reconstruction of missing pixels, and IS and FID provide insights into the generated samples’ diversity, realism, and overall quality.

4 $$RMSE=\sqrt[2]{\sum_{i=o}^{n}\frac{\:\left(genrated\:value-actual\:value\right)^2}{n}}$$

5 $$\:PSNR=10\cdot\:{\text{log}}_{10}\frac{MSE}{MA{X}^{2}}$$

6 $$\:\text{I}\text{S}=\text{e}\text{x}\text{p}(\text{E}\text{x}\sim\:\text{p}\text{g}\text{D}\text{K}\text{L}(\text{p}\left(\text{y}\right|\text{x}\left)\right|\left|\text{p}\right(\text{y}\left)\right)).$$

7 $$\:\text{F}\text{I}\text{D}=\parallel\:{\upmu\:}\text{r}-{\upmu\:}\text{g}\parallel\:2+\text{T}\text{r}({\Sigma\:}\text{r}+{\Sigma\:}\text{g}-2({\Sigma\:}\text{r}{\Sigma\:}\text{g})1/2)).$$

## Experimental results

In this case study, we used five new diverse datasets to evaluate the effectiveness of different models in missing pixel imputation and segmentation tasks. The first dataset, BigGAN-ImageNet^39^, contains high-resolution images generated by the BigGAN model and provides various classes with inherent missing pixel simulations. This dataset al.lowed us to test our model’s ability to handle diverse image characteristics and assess their robustness. The 2024 Medical Imaging Challenge Dataset^40^ comprises medical images, such as CT and MRI scans, and includes annotated missing pixel regions. We focused on how well ECP-IGANN could impute these missing data points while maintaining the integrity of critical medical features given the high stakes of accurate medical imaging. Next, we explored a Synthetic Data Generation for Autonomous Vehicles dataset^41^, which simulates driving environments with occlusions and missing pixel data. This dataset poses unique challenges due to its dynamic conditions, enabling us to examine the robustness of our models in real-world scenarios where data loss can occur. The 2024 Satellite Imagery Dataset^42^ featured satellite images affected by natural phenomena like cloud cover, providing an opportunity to assess segmentation capabilities in the context of missing pixel imputation. The complexity of these images served as a rigorous test for our methodologies, particularly in environmental monitoring. Finally, we included the Fashion and Apparel Dataset 2024^43^, which contains fashion images with instances of missing pixels. This dataset was particularly useful for exploring image completion tasks because the annotations allowed us to evaluate our model’s segmentation performance across various fashion items. To comprehensively assess the performance of the proposed model, we tested our primary model against seven other architectures, focusing on their diversity and ability to handle missing pixel imputation. Additionally, we employed various U-Net architectures to compare segmentation performance before and after applying missing pixel imputation techniques. This approach provided valuable insights into the strengths and weaknesses of different models in addressing the complexities associated with missing pixels in medical and remote sensing fields.

### Results of Pixel Imputation

Table 2 presents the pixel imputation results for ECP-IGANN and seven recent GAN models for missing pixel imputation for five different datasets in terms of RMSE. The results show the efficiency of ECP-IGANN and WGANs compared with other versions of GANs for missing data imputation regarding RMSE. They also demonstrate the outperformance of ECP-IGANN compared with different models.

The table compares RMSE values for eight models across five datasets, evaluating their effectiveness in missing data imputation. ECP-IGANN consistently outperforms the other models, achieving the lowest RMSE in all datasets, highlighting its superior accuracy in imputing missing pixels. For example, on the BigGAN-ImageNet dataset, ECP-IGANN records an RMSE of 0.061, significantly lower than the closest competitor, WGAIN, which has an RMSE of 0.073. These results demonstrate ECP-IGANN’s exceptional handling of complex and diverse image data. In the 2024 Medical Imaging Challenge Dataset, ECP-IGANN again leads with an RMSE of 0.132, outperforming GAIN and MTS-GAN, which have higher RMSEs of 0.1839 and 0.184, respectively, underscoring its capability to maintain critical image details in clinical applications. ECP-IGANN also excels in the Autonomous Vehicles dataset with an RMSE of 0.0697, highlighting its effectiveness in accurately imputing missing data in dynamic and safety-critical environments. In the 2024 Satellite Imagery Dataset, ECP-IGANN achieves an RMSE of 0.0974, outperforming other models, such as CM-GAN (0.113) and VIGAN (0.126), demonstrating its strength in managing spatial coherence and large-scale imputation tasks. Finally, on the Fashion and Apparel Dataset 2024, ECP-IGANN records the lowest RMSE of 0.0491, showing its proficiency in restoring missing details in datasets where fine textures and patterns are critical.

**Table 2:** Comparison of RMSE among seven models and ECP-IGANN across five datasets.

Model	Dataset				
	BigGAN-ImageNet	2024 Medical Imaging Challenge Dataset	Autonomous Vehicles dataset	2024 Satellite Imagery Dataset	Fashion and Apparel Dataset 2024
GAIN	0.121	0.1839	0.113	0.143	0.074
WGAIN	0.073	0.153	0.089	0.105	0.056
SpatialGAIN	0.116	0.164	0.121	0.118	0.073
VIGAN	0.102	0.153	0.133	0.126	0.079
CM-GAN	0.077	0.158	0.099	0.113	0.059
GAMIN	0.089	0.167	0.101	0.139	0.0793
MTS-GAN	0.101	0.184	0.126	0.132	0.0873
ECP-IGANN	0.061	0.132	0.0697	0.0974	0.0491

**Fig. 2:**
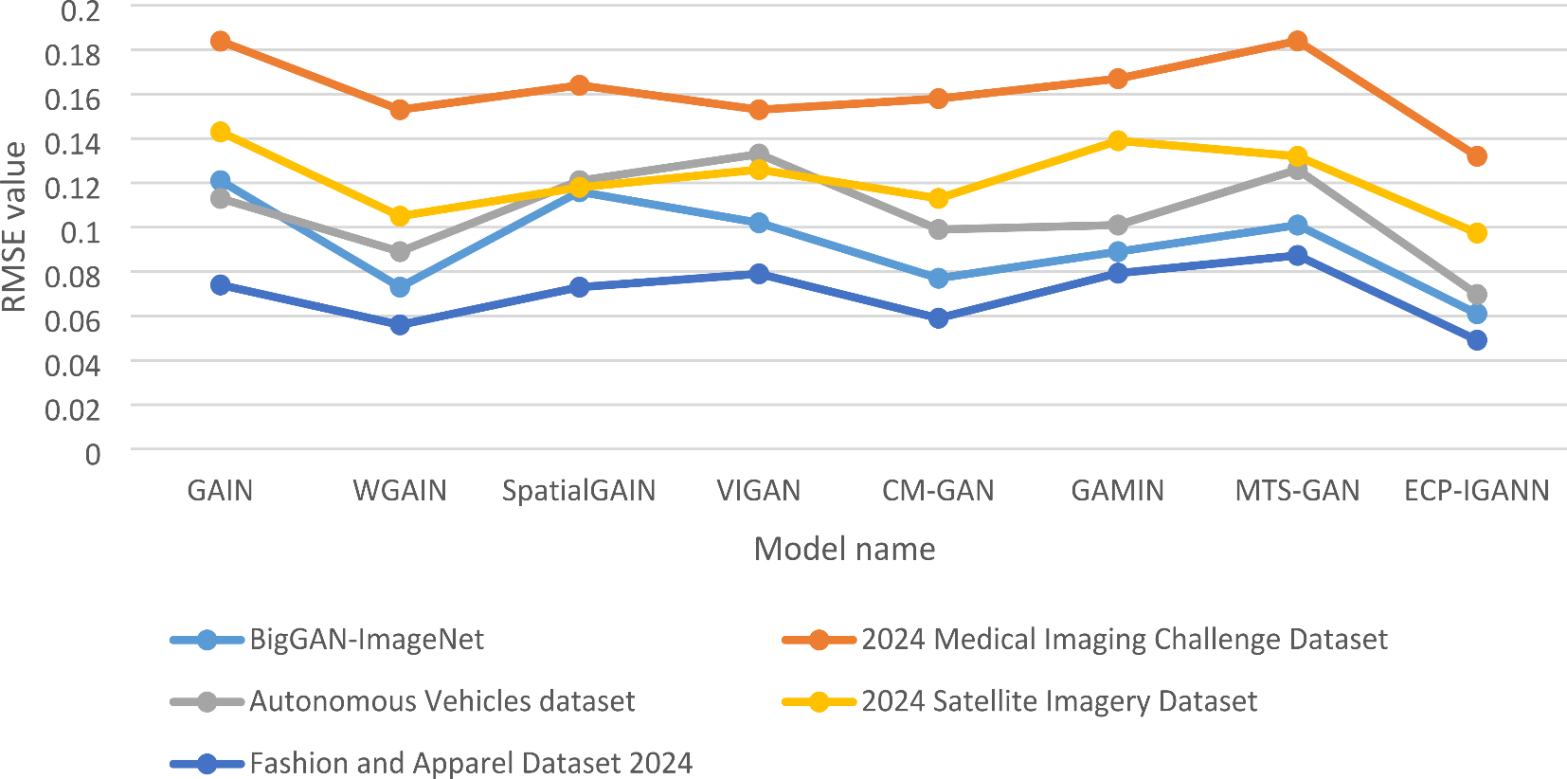
RMSE comparison across five different datasets.

Compared to other models, such as WGAIN and CM-GAN, which generally perform well, ECP-IGANN’s consistently lower RMSE across all datasets highlights its enhanced capabilities, making it a highly effective tool for missing pixel imputation in diverse applications. Figure 2 shows the outperformance of the ECP-IGANN for the seven different models.

**Fig. 3:**
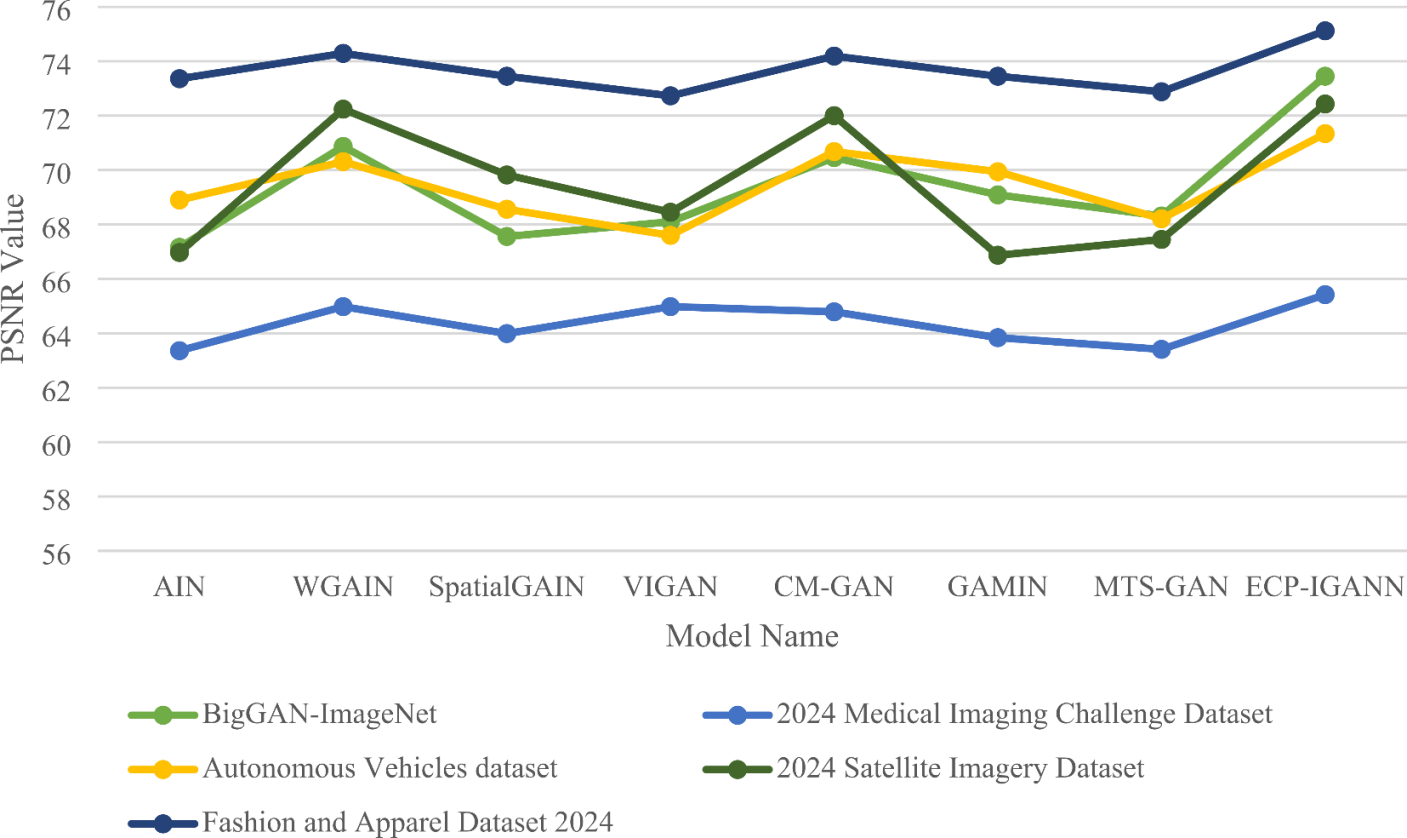
PSNR comparison across five different datasets.

Table 3 compares the Relative Signal-to-Noise Ratio (RSNR) values of seven models, including ECP-IGANN, across five datasets. The RSNR metric provides insights into image reconstruction quality, with higher values indicating better performance. In this analysis, ECP-IGANN achieves the highest RSNR values across all datasets, reaching 73.45 on the BigGAN-ImageNet dataset and 75.12 on the Fashion and Apparel Dataset 2024. These results suggest that ECP-IGANN is particularly effective at preserving image quality, making it a strong option for tasks requiring high fidelity in reconstruction. WGAIN follows closely behind, with impressive RSNR scores, particularly in the 2024 Satellite Imagery Dataset (72.24) and the Fashion and Apparel Dataset 2024 (74.29). These results indicate that WGAIN excels in crucial scenarios where capturing fine details is vital. CM-GAN demonstrates competitive performance, especially in the Autonomous Vehicles dataset (70.68) and the 2024 Satellite Imagery Dataset (72.00). The results suggest a robust capability in handling various image data types, although it does not surpass ECP-IGANN or WGAIN. GAMIN and VIGAN show moderate RSNR values, with GAMIN scoring 69.94 in the Autonomous Vehicles dataset and VIGAN achieving 68.10 in the BigGAN-ImageNet dataset. These models demonstrate reliable performance, though they lag behind the top performers. SpatialGAIN yields comparable results with an RSNR of 69.82 in the 2024 Satellite Imagery Dataset, highlighting its ability to maintain image quality through spatial context. However, it still falls short of the leading models. MTS-GAN had the lowest RSNR values among the models analyzed, particularly in the 2024 Medical Imaging Challenge Dataset (63.41). These results suggest that although it has some strengths, it may struggle with more complex data types or specific scenarios. Figure 3 presents a comparison chart between ECP-IGANN and the seven other architectures of GANs in terms of RSNR.

**Table 3:** Comparison of RSNR among seven models and ECP-IGANN across five datasets.

Model	Dataset				
	BigGAN-ImageNet	2024 Medical Imaging Challenge Dataset	Autonomous Vehicles dataset	2024 Satellite Imagery Dataset	Fashion and Apparel Dataset 2024
AIN	67.16	63.36	68.90	66.97	73.36
WGAIN	70.87	64.98	70.31	72.24	74.29
SpatialGAIN	67.56	63.99	68.56	69.82	73.45
VIGAN	68.10	64.98	67.60	68.45	72.73
CM-GAN	70.46	64.79	70.68	72.00	74.19
GAMIN	69.09	63.84	69.94	66.87	73.45
MTS-GAN	68.31	63.41	68.21	67.45	72.88
ECP-IGANN	73.45	65.42	71.34	72.43	75.12

### Generation diversity results

**Fig. 4:**
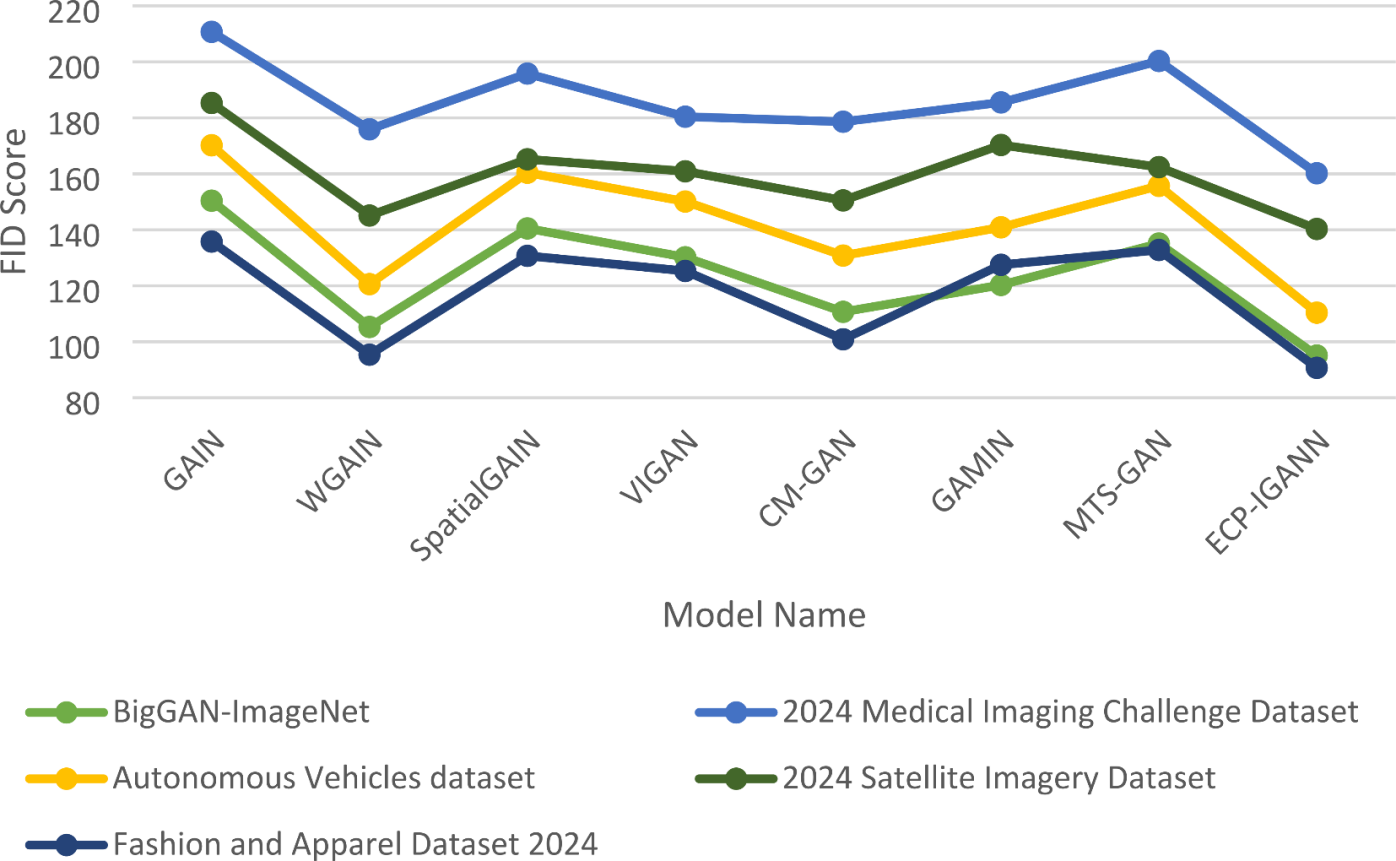
FID comparison across five different datasets.

The comparison of FID (Fréchet Inception Distance) and IS (Inception Score) values across various models highlights the superior performance of ECP-IGANN in generating high-quality and diverse images. As shown in Table 4, ECP-IGANN consistently achieved the lowest FID scores across all datasets, including BigGAN-ImageNet, the 2024 Medical Imaging Challenge, Autonomous Vehicles, 2024 Satellite Imagery, and the Fashion and Apparel Dataset 2024. These low FID scores indicate that ECP-IGANN excels at generating high-quality images and minimal artifacts, effectively preserving pixel relationships and ensuring that the generated images closely resemble accurate data. In comparison, other models like WGAIN and CM-GAN also performed well but still exhibited higher FID values, suggesting slightly less effective image generation, particularly in more complex datasets. Models such as GAIN and MTS-GAN exhibited higher FID scores, reflecting difficulties in maintaining image quality and coherence (Fig. 4).

**Table 4:** Comparison of FID among seven models and ECP-IGANN across four datasets.

Model	Dataset				
	BigGAN-ImageNet	2024 Medical Imaging Challenge Dataset	Autonomous Vehicles dataset	2024 Satellite Imagery Dataset	Fashion and Apparel Dataset 2024
GAIN	150.4	210.7	170.2	185.3	135.8
WGAIN	105.3	175.9	120.6	145.1	95.4
SpatialGAIN	140.5	195.8	160.4	165.2	130.7
VIGAN	130.2	180.4	150.1	160.9	125.3
CM-GAN	110.7	178.6	130.8	150.5	100.9
GAMIN	120.3	185.5	140.9	170.3	127.5
MTS-GAN	135.1	200.3	155.7	162.4	132.8
ECP-IGANN	95.1	160.2	110.4	140.3	90.7

**Fig. 5:**
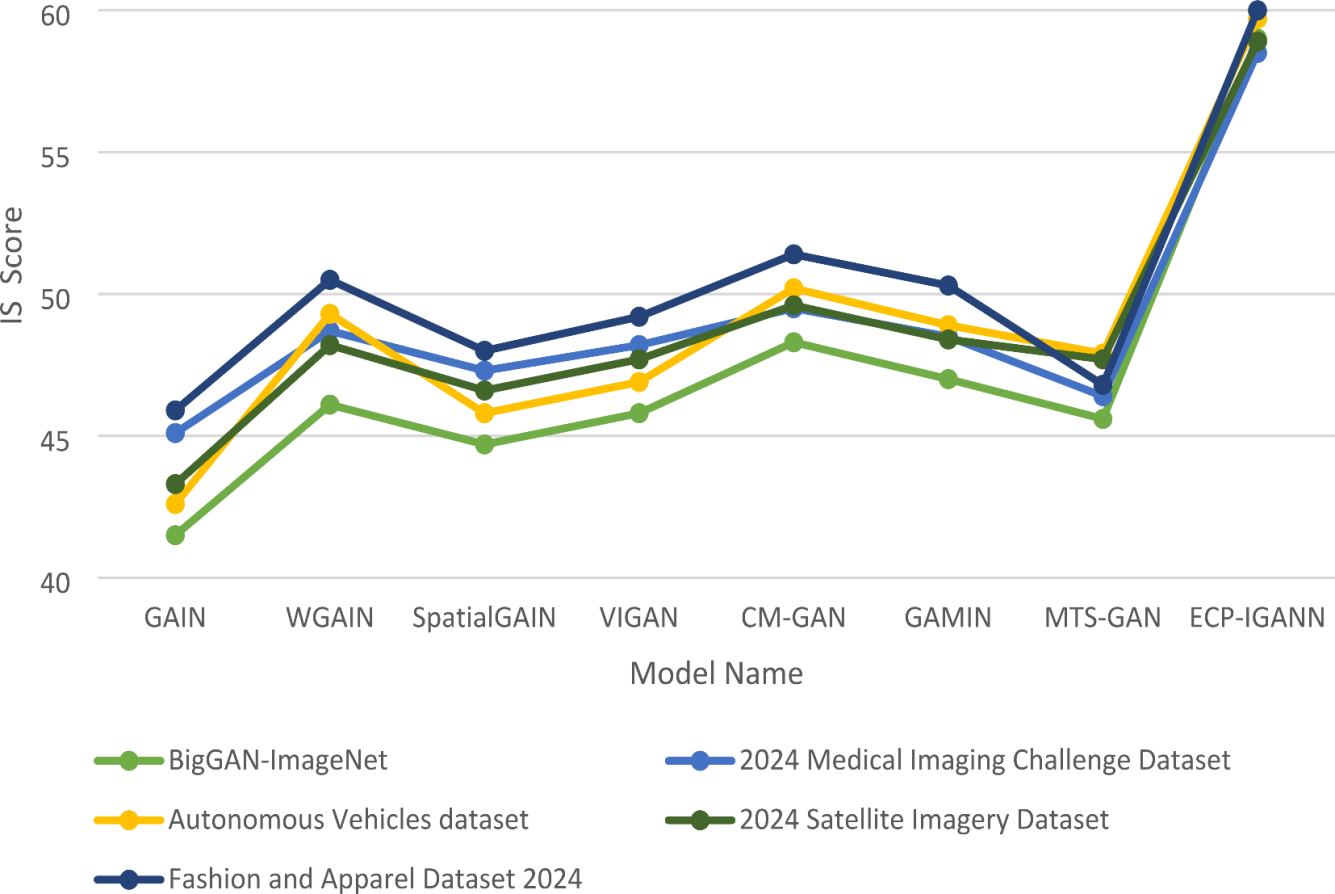
IS comparison across four different datasets.

Table 5 demonstrates ECP-IGANN’s dominance with the highest IS values across all five datasets, reflecting its exceptional ability to generate diverse and visually convincing images. The IS values of ECP-IGANN range from 58.5 to 60.0, demonstrating high image quality and a wide variety of generated outputs. These results contrast the low IS scores observed in models with difficulty achieving the same degree of diversity and realism, such as GAIN, SpatialGAIN, and MTS-GAN. Although CM-GAN and WGAIN perform better, there IS scores still fall short of ECP-IGANN’s, reaffirming the latter’s superior capability in handling complex image imputation tasks. As illustrated in Fig. 5, these results position ECP-IGANN as the leading model, providing the most effective balance of image quality, diversity, and accuracy across various challenging datasets.

**Table 5:** Comparison of IS among seven models and ECP-IGANN across five datasets.

Model	Dataset				
	BigGAN-ImageNet	2024 Medical Imaging Challenge Dataset	Autonomous Vehicles dataset	2024 Satellite Imagery Dataset	Fashion and Apparel Dataset 2024
GAIN	41.5	45.1	42.6	43.3	45.9
WGAIN	46.1	48.7	49.3	48.2	50.5
SpatialGAIN	44.7	47.3	45.8	46.6	48.0
VIGAN	45.8	48.2	46.9	47.7	49.2
CM-GAN	48.3	49.5	50.2	49.6	51.4
GAMIN	47.0	48.5	48.9	48.4	50.3
MTS-GAN	45.6	46.4	47.9	47.7	46.8
ECP-IGANN	59.0	58.5	59.7	58.9	60.0

Table 6 compares the performance of various GAN-based models on different datasets, specifically evaluating their applicability to the BigGAN-ImageNet, 2024 Medical Imaging Challenge, Autonomous Vehicles, 2024 Satellite Imagery, and Fashion and Apparel 2024 datasets. The models considered are GAIN, WGAIN, SpatialGAIN, VIGAN, CM-GAN, GAMIN, MTS-GAN, and ECP-IGANN. Among these models, the WGAIN, CM-GAN, and ECP-IGANN consistently demonstrate successful applicability across all datasets, indicating their robust and generalized capabilities for different data types. In contrast, VIGAN demonstrated high applicability but fell short for the Autonomous Vehicles dataset, suggesting a limitation in handling data types specific to this domain. GAIN, SpatialGAIN, GAMIN, and MTS-GAN are uniformly marked as “False” across all datasets, indicating their lack of effectiveness or applicability in these scenarios. This consistent non-applicability highlights potential architectural shortcomings or adaptability to the diverse and complex data presented by these specific datasets.

**Table 6:** Mode collapse mitigation results of various models.

Model	Dataset				
	BigGAN-ImageNet	2024 Medical Imaging Challenge Dataset	Autonomous Vehicles dataset	2024 Satellite Imagery Dataset	Fashion and Apparel Dataset 2024
GAIN	False	False	False	False	False
WGAIN	True	True	True	True	True
SpatialGAIN	False	False	False	False	False
VIGAN	True	True	False	True	True
CM-GAN	True	True	True	True	True
GAMIN	False	False	False	False	False
MTS-GAN	False	False	False	False	False
ECP-IGANN	True	True	True	True	True

### Results of the segmentation process

The experimental results highlight the effectiveness of ECP-IGANN in improving segmentation performance across different datasets and models. Initially, the segmentation results before applying ECP-IGANN, as shown in Tables 7 and 8, indicate that models such as Spatial Attention U-Net, Dense U-Net, and Residual Attention U-Net achieved respectable but varied performance in terms of Dice Score, Accuracy, Precision, and Recall on both the 2024 Medical Imaging Challenge Dataset and the 2024 Satellite Imagery Dataset. For example, Residual Attention U-Net demonstrated the highest Dice Score and Accuracy across both datasets, emphasizing its ability to handle complex segmentation tasks.

**Table 7:** Segmentation results prior to applying ECP-IGANN for the 2024 Medical Imaging Challenge dataset.

Model	Dice Score ↑	Accuracy ↑	Precision ↑	Recall ↑
Spatial Attention U-Net^44^	0.82	0.87	0.84	0.81
Dense U-Net^45^	0.81	0.86	0.83	0.80
Feature Pyramid U-Net^46^	0.79	0.85	0.81	0.78
Channel Attention U-Net^47^	0.80	0.85	0.82	0.79
Residual Dense U-Net^48^	0.83	0.88	0.85	0.82
Residual Attention U-Net^49^	0.84	0.88	0.86	0.83

**Table 8:** Segmentation results after applying ECP-IGANN for the 2024 Medical Imaging Challenge dataset.

Model	Dice Score ↑	Accuracy ↑	Precision ↑	Recall ↑
Spatial Attention U-Net^44^	0.88	0.91	0.89	0.87
Dense U-Net^45^	0.87	0.90	0.88	0.86
Feature Pyramid U-Net^46^	0.85	0.89	0.87	0.84
Channel Attention U-Net^47^	0.86	0.89	0.88	0.85
Residual Dense U-Net^48^	0.89	0.92	0.90	0.88
Residual Attention U-Net^49^	0.90	0.93	0.91	0.89

However, after integrating ECP-IGANN for missing pixel imputation, there was a noticeable enhancement in the segmentation metrics, as presented in Tables 9 and 10. The Dice Scores, Accuracy, Precision, and Recall values for all models improved significantly, with the Residual Attention U-Net reaching a Dice Score of 0.90 and an Accuracy of 0.93 on the Medical Imaging dataset, and similar improvements were observed in the Satellite Imagery dataset. These results validate the hypothesis that the superior pixel imputation provided by ECP-IGANN leads to more coherent and accurate image reconstruction, thereby improving downstream tasks like segmentation.

This comprehensive validation across multiple models and datasets demonstrates the robust applicability of ECP-IGANN. Consistent improvements across all metrics—Dice Score, Accuracy, Precision, and Recall—underscore the model’s potential to enhance performance in various challenging imaging domains, making it a valuable tool in medical and remote sensing applications.

**Table 9:** Segmentation results prior to applying ECP-IGANN for the 2024 Satellite Imagery dataset.

Model	Dice score ↑	Accuracy ↑	Precision ↑	Recall ↑
Spatial Attention U-Net^44^	0.79	0.84	0.80	0.78
Dense U-Net^45^	0.78	0.83	0.79	0.77
Feature Pyramid U-Net^46^	0.76	0.82	0.77	0.75
Channel Attention U-Net^47^	0.77	0.82	0.78	0.76
Residual Dense U-Net^48^	0.80	0.85	0.81	0.79
Residual Attention U-Net^49^	0.81	0.86	0.82	0.80

**Table 10:** Segmentation results after applying ECP-IGANN for the 2024 Satellite Imagery dataset.

Model	Dice Score ↑	Accuracy ↑	Precision ↑	Recall ↑
Spatial Attention U-Net^44^	0.85	0.89	0.86	0.84
Dense U-Net^45^	0.84	0.88	0.85	0.83
Feature Pyramid U-Net^46^	0.82	0.87	0.83	0.81
Channel Attention U-Net^47^	0.83	0.87	0.84	0.82
Residual Dense U-Net^48^	0.86	0.90	0.87	0.85
Residual Attention U-Net^49^	0.87	0.91	0.88	0.86

## Conclusions

In this paper, we have proposed an 8-connected Pixel Identity GAN with Neutrosophic (ECP-IGANN) to address the challenges in missing pixel imputation. The proposed method enhances the traditional GAN architecture by introducing two key innovations: integrating an identity block into the generator and applying a neutrosophic-based loss function that evaluates the spatial coherence of the generated pixels. The identity block plays a crucial role in preserving the essential features of the original image during the generation process. By allowing a parallel flow of information from input to output, the identity block ensures that the generated pixels retain critical spatial relationships and do not deviate from the original content. This addressing helps mitigate the mode collapse problem, where the generator typically produces limited and repetitive outputs. The neutrosophic-based 8-connected pixel loss function is another significant contribution of this work. This loss function goes beyond simple pixel-wise comparison by evaluating the coherence of each pixel within its 8-connected neighbourhood. By leveraging neutrosophic logic, which accounts for truth, indeterminacy, and falsehood in pixel values, the loss function provides a more nuanced and accurate assessment of the pixels in images generated by GANs with identity block. This integration ensures that the imputed pixels match the surrounding context and maintain the overall spatial integrity of the generated image. Experiments were conducted using new datasets, and the results proved the model’s efficiency in diversity, missing imputation, and mode collapse mitigation. The results also demonstrate the efficiency of using the model as a preprocessing step for the different architectures of U-Nets in enhancing the segmentation accuracy.

Although ECP-IGANN demonstrates superior performance in missing pixel imputation, it has certain limitations. Including the identity block and neutrosophic loss function increases the computational complexity of the model, which may result in longer training times and higher resource consumption. Future research could focus on optimizing the computational efficiency of ECP-IGANN, perhaps by simplifying the architecture or exploring alternative loss functions that maintain performance while reducing complexity. Another avenue for future work could be the adaptation of ECP-IGANN for different types of data, such as temporal data (e.g., video frames) or volumetric data (e.g., 3D medical images), where the spatial coherence of generated pixels is equally crucial.

## Data Availability

The datasets and code generated during the current study are available from the corresponding author upon reasonable request.
